# Supplementary material for: Dynamics of growing carbon nanotube interfaces probed by machine learning-enabled molecular simulations
Source: Nat Commun. 2024 May 14;15:4076. doi: 10.1038/s41467-024-47999-7 (PMC11094095; doi:10.1038/s41467-024-47999-7)
Supplement: Supplementary file 1 — Supplementary Information [file 41467_2024_47999_MOESM1_ESM.pdf]

Supplementary Information for

Dynamics of growing carbon nanotube interfaces probed by machine learning-enabled molecular simulations

Daniel Hedman, Ben McLean, Christophe Bichara, Shigeo Maruyama, J. Andreas Larsson and Feng Ding

Corresponding authors: Daniel Hedman, daniel.hedman@ltu.se and Feng Ding, f.ding@siat.ac.cn

Here, additional information is presented to support the results in the main text. This includes evaluation of the accuracy of the DeepCNT-22 machine learning force field (MLFF), details of the five phases of nanotube growth, derivation of the probability density function, cumulative distribution function and expected value of τ. As well as derivation of a straightforward model to predict $\langle N_{\mathrm{C}} \rangle$ and the impact of surface-adsorbed hydrogen on the carbon-metal adhesion energy.

1. Validation of DeepCNT-22

Validating the accuracy of MLFFs is a crucial step for which multiple methods have been proposed [1]. In this case, the energy and force accuracy of DeepCNT-22 is evaluated on a subset of structures, constituting 10% of the entire dataset shown in Fig. 1 of the main text. On this test set (not used in the training process), the root mean square error (RMSE) of the energy and force predictions from DeepCNT-22 was calculated.

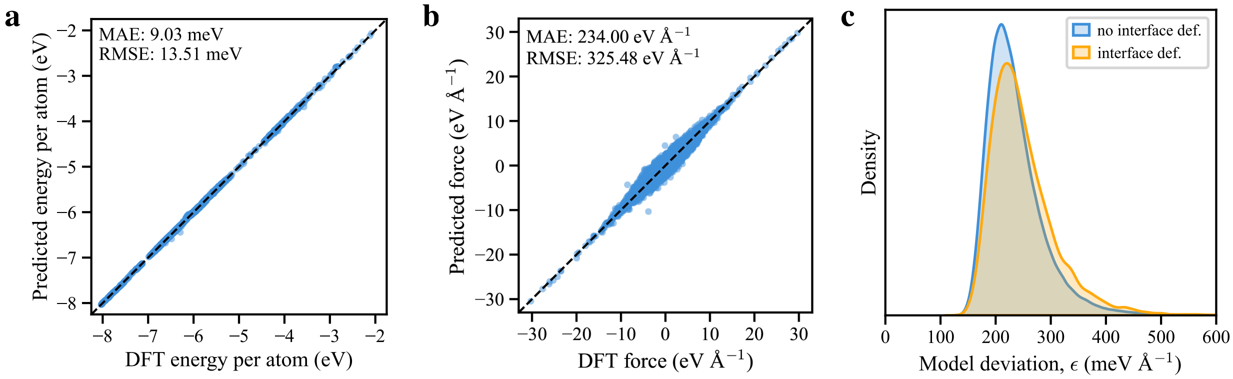

Fig. S1. Regression plots for the **a** energy and **b** force predictions of DeepCNT-22 evaluated on the density functional theory (DFT) test data. **c** kernel density estimate (KDE) of the model deviation during growth of the (6,5) single-walled carbon nanotube for tube-catalyst interfaces with defects (orange) and without (blue). The sample size used to calculate the mean absolute error (MAE) and root mean square error (RMSE) in **a** and **b** were 2297 energies and 229,012 forces components respectively. For **c**, the sample size used to fit the KDE was 378,303 timesteps for tube-catalyst interfaces without defects (blue) and 19,243 for those with defects (orange). Source data for this figure is provided in the Source Data file.

The regression plots in Fig. S1a and 1b show that DeepCNT-22 can reproduce the density functional theory (DFT) energies and forces of the test dataset with an RMSE of 13.51 meV per atom

for energies and 325.48 meV/Å for forces, comparable to other published MLFFs based on the same architecture [2, 3].

In addition to evaluating DeepCNT-22 on a test data set, the accuracy of the MLFF was continuously evaluated during each single-walled carbon nanotube (SWCNT) growth simulation using the model deviation, ϵ. [4] As shown in Fig. S1c the distribution of ϵ for timesteps with interface defects (orange) and those without (blue) show only a single peak centered around 250 meV/Å respectively. DeepCNT-22 thus maintains accuracy throughout the growth simulations and shows no bias towards defective or defect-free growth.

Apart from validating the accuracy in energy and forces of DeepCNT-22, it is essential to ensure that the MLFF can reproduce known physical properties of the target system. To this end, DeepCNT-22's ability to reproduce the subtle energy difference of SWCNTs with varying curvature, i.e., the SWCNT curvature energy, E_c, is examined. Previous studies [5] have shown that this energy can be described as a function of the curvature, $1/R$, of a SWCNT of radius R as $E_c = \gamma \cdot (1/R)^2$. Here a value of $\gamma = 2.038$ eVÅ^2 obtained via previous DFT calculations [6] was used. As shown in Fig. S2a, DeepCNT-22 can reproduce the curvature energy of SWCNTs over a wide diameter range with high accuracy.

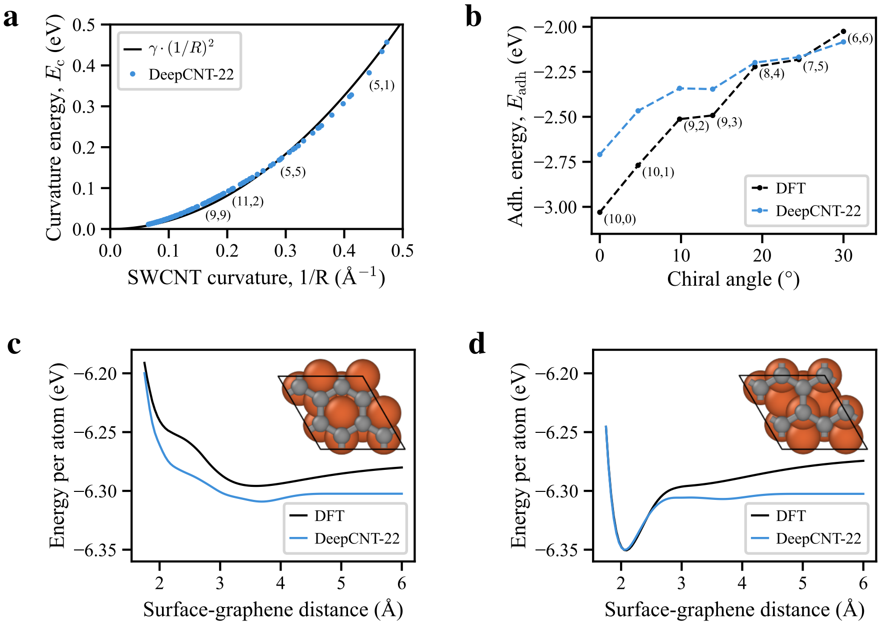

Fig. S2. Validation of DeepCNT-22's ability to accurately reproduce properties related to growth. **a** the curvature energy for single-walled carbon nanotubes (SWCNTs), computed with DeepCNT-22, compared to the known analytical expression fitted to density functional theory (DFT) calculations. **b** the carbon-metal adhesion energy of SWCNTs attached to "clean" iron clusters, calculated using both DeepCNT-22 and DFT. **c** and **d** the 1D potential energy surface of graphene adsorbed on an Fe(111) surface in the fcc–hollow and fcc-top configurations, respectively.

DeepCNT-22's ability to reproduce the carbon-metal adhesion energy [7–9] of SWCNTs with various chiralities attached to Fe_{55} clusters was also evaluated. The adhesion energy of a SWCNT with chirality n, m is calculated as $E^{n,m}_{\text{adh}} = \left(E^{n,m}_{\text{cx}} - \left(E_{\text{Fe}_{55}} + E^{n,m}\right)\right)/(n+m)$, where $E^{n,m}_{\text{cx}}$ is the energy of the complex (SWCNT attached to a Fe_{55} cluster), $E_{\text{Fe}_{55}}$ is the energy of the cluster itself, and $E^{n,m}$ is the energy of the SWCNT. As shown in Fig. S2b, DeepCNT-22 can reproduce the trend in the carbon-metal adhesion energy as a function of the tube's chirality. While an exact agreement

with DFT is not reached, DeepCNT-22's ability to reproduce this trend is noteworthy, given that the training data does not contain structures of SWCNTs attached to "clean" Fe clusters, i.e., clusters with no carbon atoms dissolved, as well as no structures of open-ended SWCNTs. This indicates that DeepCNT-22 can generalize reasonable well to structures outside of the training data.

An important property for SWCNT growth is the adhesion strength between graphitic carbon and the metal surface, as it affects SWCNT-cap lift off versus encapsulation of the catalyst [10]. Fig. S2c and 2d shows the 1D potential energy surface of graphene adsorbed on an Fe(111) surface in the fcc–hollow and fcc-top configurations, respectively. As shown, DeepCNT-22 can reproduce both the shallow minimum for the fcc–hollow and the deeper minimum for the fcc-top configuration. It is important to note that the adhesion energy for DeepCNT-22 plateaus at around 5.0 Å, as this is the cutoff used for the MLFF.

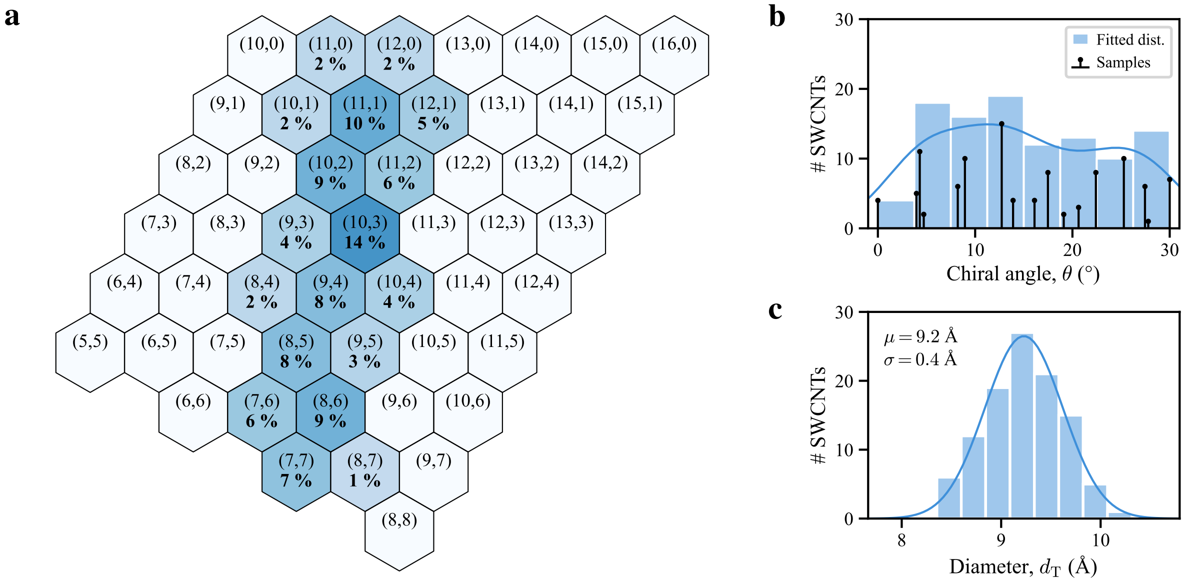

Fig. S3. Chirality distribution of 106 single-walled carbon nanotubes (SWCNTs) grown using Fe_{55} catalysts at a growth temperature of $T = 1300$ K and a carbon supply rate of $k = 0.5$ ns^{-1}. **a** the chirality map showing the abundance of each chirality (n, m) observed post-growth. **b** and **c** show the distributions of the chiral angle and SWCNT diameter, respectively. The sample size used to derive statistics in **a**, **b**, and **c** was 106 tubes.

Experiments have shown that when SWCNTs are grown using Fe catalysts and simple hydrocarbon feedstock gas, the final product exhibits a broad chirality distribution [11, 12]. To evaluate DeepCNT-22's ability to accurately describe the nucleation of SWCNTs, where chirality is set, 280 growth simulations were conducted under identical conditions, Fe_{55} catalyst, 1300K growth temperature, and 200 carbon atoms supplied at a rate of $k = 0.5$ ns^{-1}. These simulations resulted in 106 SWCNTs with well-defined chirality which are shown in Fig. S7, corresponding to a yield of 37.9%. The remaining 174 SWCNTs, with undefinable chirality, are shown in Fig. S8. Here, a variety of failure modes can be seen such as. "Cap liftoff failure" where the SWCNT-cap fails to lift from the catalyst causing encapsulation. "Cap formation failure" where a conical cap is formed which causes the diameter of the graphite structure to continue to increase/decrease until a size limit is reached, as determined by the size of the catalyst. "Defect induced failure" where an interface defect becomes trapped inside the tube wall during growth causing a change in chirality. Note that all of these failure modes are the result of an inappropriate number of penta- or heptagons

forming the graphite structure. A broad chirality distribution is expected for these growth conditions, and as observed in the chiral map in Fig. S3a and in the distribution of the chiral angles in Fig. S3b this is indeed the case for SWCNTs grown with DeepCNT-22. Impressively, the diameter distribution of the grown tubes, as shown in Fig. S3c, agrees well with the experimental results of Ago et al. [13], who grew SWCNTs on Fe catalysts of size 1.5-3 nm.

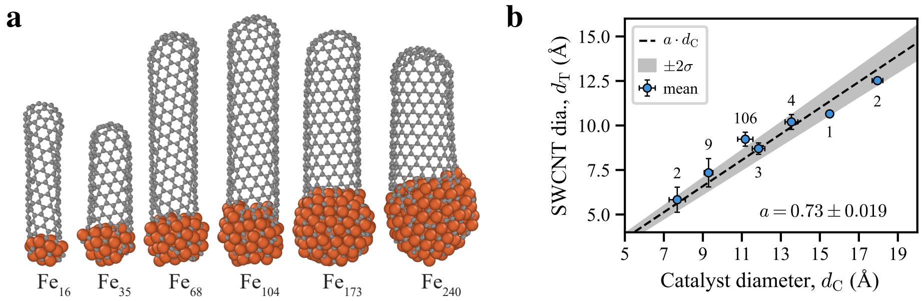

Fig. S4. Single-walled carbon nanotubes (SWCNTs) grown on Fe catalysts with varying diameters, d_C. **a** shows an example of typical structures obtained after growth. **b** are resulting tube diameters, d_T, where the data points show the mean SWCNT diameter with the error bars denoting the standard deviation. d_T follows a linear relationship $d_T = a \cdot d_C$ with $a = 0.73 \pm 0.019$ as shown by the dashed line where grey bands indicate 2σ confidence intervals of the fit. The number of SWCNTs grown for each catalyst size (sample size) is marked above the data points.

Like the chirality distribution, another property of growth is the ratio between the diameter of the grown tube and that of the catalyst. Previous growth experiments [14, 15] and theoretical studies [16, 17] have established a link between the tube diameter and the catalyst diameter, where the tube diameter is between 0.7-1.0 times the catalyst diameter. Fig. S4a displays a selection of tubes grown using DeepCNT-22 on Fe catalysts of varying sizes, where it is evident that the diameter of the tube increases with the diameter of the catalyst. To quantify this, multiple growth simulations where performed on catalysts of different sizes. Here the growth temperature was set to $T = 1300$ K for each catalyst size, but the total number of carbon atoms added and the supply rate, k, was adjusted for each catalyst size to achieve growth. Fig. S4b illustrates the mean SWCNT diameter as a function of the mean catalyst diameter, where a linear relationship can be found $d_T = a \cdot d_C$, with $a = 0.73 \pm 0.019$, in agreement with previous studies.

2. The five phases of nanotube growth

The growth process of the $(6, 5)$ SWCNT can be observed in Fig. 2A of the main text and Supplementary Movie 1, revealing the following. In the earliest (1st) phase of growth, monomers and dimers are the dominant carbon species. This observation is supported by the snapshot at $t = 8.80$ ns and the carbon species analysis presented in Fig. 2B of the main text, indicating that almost no carbon chains are present on the catalyst during the 1st phase. Notably, the high carbon supply rate employed at the beginning of growth, $k = 5$ ns^{-1}, leads to a significantly shorter 1st phase compared to using a slower rate of $k = 0.5$ ns^{-1}.

The 2nd phase of growth is characterized by the transformation of carbon monomers and dimers into linear carbon chains, as seen in the snapshot structure at $t = 28.4$ ns and the steady increase in the number of carbon atoms within chains, as shown in Fig. 2B. After reaching a critical ratio of

approximately 1/3 carbon atoms as part of chains, the growth enters the 3rd phase. Here, a junction forms on a long chain when a monomer, dimer, or the end of another chain attaches, creating a three-coordinated carbon atom. This action causes the resulting chain-like structure to fold and form the first carbon ring, as seen in the snapshot at $t = 34.25$ ns. The creation of the first carbon ring facilitates rapid subsequent ring formation, evidenced by the sharp decrease in the number of atoms in chains and the simultaneous sharp increase in the number of graphitic carbons shown in Fig. 2B. As a result, linear carbon chains are almost entirely eliminated (Fig. 2B) as they become part of the growing graphitic structure seen at $t = 39.60$ ns.

Following this, the growth enters the 4th phase, in which the graphitic structure enlarges as carbon monomers and dimers attach to its edge. As the graphitic structure expands, its curvature increases, and due to the energetic advantage of a near-perpendicular interface between the catalyst and the graphitic structure [10], it lifts off the catalyst. The SWCNT-cap is fully defined once at least six pentagons form inside the graphitic structure [18], which occurs at $t = 132.41$ ns. Subsequently, the growth enters the 5th and final phase, characterized by the continuous elongation of the tube through the attachment of carbon atoms at its edge.

3. Derivation of the PDF, CDF and expected value of τ

As observed in Fig. 1F of the main text τ follows a power-law distribution ($\tau^{-\alpha}$) with an exponential cutoff ($e^{-\lambda_2 \tau}$). This distribution's probability density function (PDF) can be expressed as

$$f_\tau(\tau) = C \cdot \tau^{-\alpha} \cdot e^{-\lambda_2 \tau} \tag{S1}$$

and must satisfy the following normalization constraint

$$\int_{\tau_{\min}}^{\infty} C \cdot \tau^{-\alpha} \cdot e^{-\lambda_2 \tau} \, d\tau = 1. \tag{S2}$$

This constraint leads to a normalization constant of

$$C = \frac{1}{\int_{\tau_{\min}}^{\infty} \tau^{-\alpha} \cdot e^{-\lambda_2 \tau} \, d\tau} = \frac{\lambda_2^{1-\alpha}}{\Gamma\left(1-\alpha, \lambda_2 \tau_{\min}\right)}, \tag{S3}$$

here $\Gamma\left(1-\alpha, \lambda_2 \tau_{\min}\right)$ is the upper incomplete gamma function. From the PDF defined in Eq. (S1), the cumulative distribution function (CDF) can then be determined

$$F_\tau(\tau) = \int_{\tau_{\min}}^{\tau} f_\tau(x) \, dx = 1 - \frac{\Gamma\left(1-\alpha, \lambda_2 \tau\right)}{\Gamma\left(1-\alpha, \lambda_2 \tau_{\min}\right)}. \tag{S4}$$

Lastly, the expected value for τ can be determined using Eq. (S1)

$$\langle \tau \rangle = \int_{\tau_{\min}}^{\infty} \tau f_\tau(\tau) \, d\tau = \frac{1}{\lambda_2} \frac{\Gamma\left(2-\alpha, \lambda_2 \tau_{\min}\right)}{\Gamma\left(1-\alpha, \lambda_2 \tau_{\min}\right)}. \tag{S5}$$

4. Derivation of $\langle N_{\mathrm{D}} \rangle$ and $\langle N_{\mathrm{C}} \rangle$

As mentioned in the main text a straightforward model is proposed to determine the expected length of a carbon nanotube (CNT) in terms of the number of carbon atoms, $\langle N_C \rangle$, that can be reached

during growth before an interface defect is likely to become trapped in the tube wall. Assume that n_6 hexagons need to form at the interface of the growing tube to trap an interface defect. Given a growth rate, k, the average formation rate of hexagons during growth equals $k_6 = k/2$, as shown in Fig. 2C of the main text. Consequently, the critical time that an interface defect must live to become trapped inside the tube wall can be expressed as

$$\tau_c = \frac{n_6}{k_6} = \frac{2n_6}{k}. \tag{S6}$$

Since the CDF, F_τ, for the interface defect lifetimes is known, Eq. (S4), the probability, p, that an interface defect lives for at least time τ_c can be calculated using the survival function

$$p = S_\tau(\tau_c) = 1 - F_\tau(\tau_c) = \frac{\Gamma(1-\alpha,\ \lambda_2\tau_c)}{\Gamma(1-\alpha,\ \lambda_2\tau_{\min})}. \tag{S7}$$

With the probability, p, it becomes possible to calculate how many interface defects are expected to be created before one has a lifetime of at least τ_c

$$\langle N_\mathrm{D}\rangle = \frac{1}{p} = \frac{\Gamma(1-\alpha,\ \lambda_2\tau_{\min})}{\Gamma(1-\alpha,\ \lambda_2\tau_c)}. \tag{S8}$$

As shown in Fig. S5, the lifetime of interface defects, τ, does not depend on the interval between their formation, δt.

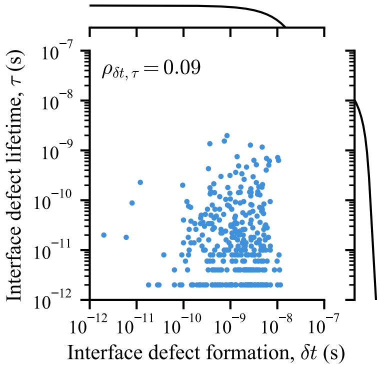

Fig. S5. The correlation between the lifetime of interface defects, τ, and their formation interval, δt. Here, the data points represent individual interface defects (one value of δt and τ given for each interface defect), the black lines show their respective probability density functions Eq. (1) and (2) of the main text and $\rho_{\delta t,\tau}$ denotes the Pearson correlation coefficient.

Given that the supply rate of carbon atoms, k, is known, it is thus possible to calculate the expected number of carbon atoms that will be added to the growing tube before an interface defect becomes trapped

$$\langle N_\mathrm{C}\rangle = \langle N_\mathrm{D}\rangle \cdot \langle \delta t\rangle \cdot k = \frac{k}{\lambda_1}\frac{\Gamma(1-\alpha,\ \lambda_2\tau_{\min})}{\Gamma\left(1-\alpha,\ \lambda_2\frac{2n_6}{k}\right)}. \tag{S9}$$

Now to calculate $\langle N_C\rangle$ using Eq. (S9), an initial value for n_6 must be assumed. Considering the worst-case scenario, in which an interface defect is formed such that only one hexagon needs

to be added to the interface of the growing tube to trap the defect, $n_6 = 1$ can be assumed. This assumption can be verified using Eq. (S8) with parameter values for the interface defect formation and lifetime distributions obtained from the growth of the $(6, 5)$ SWCNT shown in Fig. 2 of the main text, $\lambda_1 = 1.08 \cdot 10^9$ s^{-1}, $\alpha = 1.20$, $\lambda_2 = 1.04 \cdot 10^9$ s^{-1}, $\tau_{\min} = 1.10 \cdot 10^{-12}$ s, and $k = 0.5$ ns^{-1}. This yields $\langle N_D \rangle \approx 6000$ interface defects expected to form before one gets trapped inside the tube wall, significantly more than the 804 interface defects observed during growth, Sim. 1 in Table 1 of the main text. For a carbon supply rate of $k = 5$ ns^{-1}, ten times the value used to grow the $(6, 5)$ SWCNT, $\langle N_D \rangle \approx 20$ and $\langle N_C \rangle \approx 90$ are obtained. Which demonstrates that at these high growth rates, interface defects are likely to live long enough to get trapped inside the tube wall, resulting in a defective CNT. This is consistent with simulations at similar growth rates, which yielded defective tubes, and confirms that $n_6 = 1$ is a reasonable assumption. It should be noted that Eq. (S9) with $n_6 = 1$ is identical to Eq. (3) in the main text.

5. Impact of adsorbed hydrogen on the carbon-metal adhesion energy

A series of DFT calculations were performed to explore the impact of adsorbed hydrogen on the carbon-metal adhesion energy of $(6, 6)$ and $(10, 0)$ SWCNTs. Here different amounts of hydrogen were adsorbed onto an Fe_{55} cluster, and the resulting carbon-metal adhesion energies were determined following the same method detailed in Section 1. Three distinct hydrogen concentrations on the cluster surface were investigated; high (37 H atoms), medium (18 H atoms), and low (9 H atoms). For the high concentration, one hydrogen atom was adsorbed at the top site of each exposed Fe atom on the cluster surface. For the medium and low hydrogen concentrations, three different positions of the adsorbed hydrogen were tested; randomly distributed over the surface (random), close to the tube-catalyst interface (interface) and far away from the interface (bottom). All structures were then relaxed until the maximum force on each atom was less than 0.01 eV/Å, using the conjugate gradient algorithm and the same DFT parameters as described in the methods section of the main text.

During relaxation, hydrogen atoms move from the top sites to the hollow sites as seen Fig. S6a. Notably, hydrogen atoms initially placed near the interface move to sites further from the interface during relaxation, thereby not interfering with the Fe-C bonds. As shown in Fig. S6b, the carbon-metal adhesion energy for both armchair and zigzag bonds are not significantly affected by the adsorption of hydrogen onto the catalyst. Even at the highest concentration, the adhesion energy is only marginally higher than that observed for SWCNTs attached to ”clean” iron clusters. Moreover, the position of the adsorbed hydrogen does not notably influence the carbon-metal adhesion energy, as shown in Fig. S6c. Thus, it is reasonable to conclude that the small amount of hydrogen adsorbed on the iron cluster during growth does not significantly affect the Fe-C bonds at the interface.

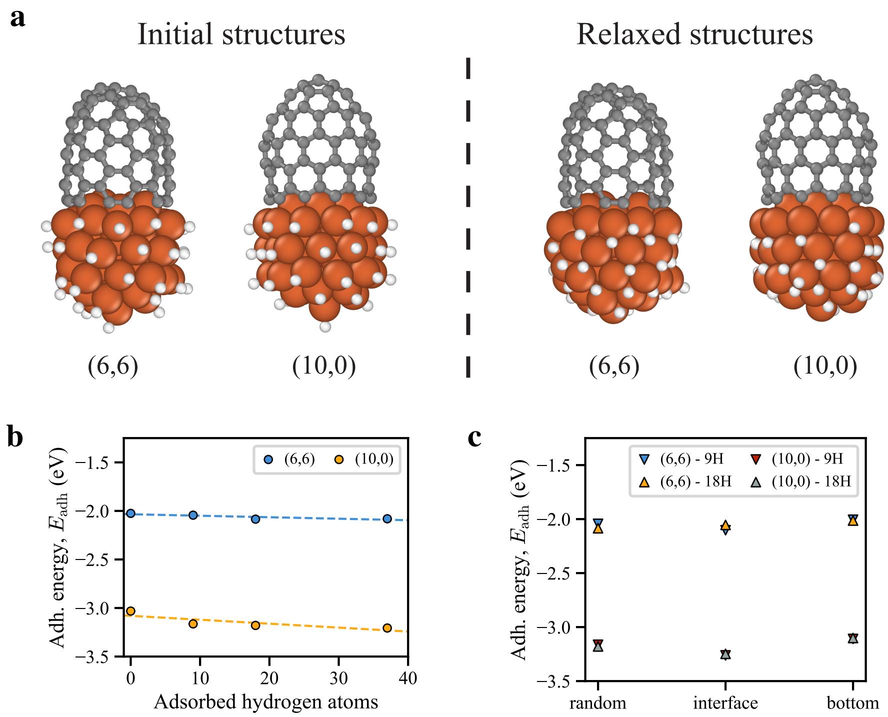

Fig. S6. The impact of adsorbed hydrogen on the carbon-metal adhesion energies for $(6,6)$ and $(10,0)$ single-walled carbon nanotubes (SWCNTs). **a** the structure before and after relaxation for the high concentration, 37 H atoms adsorbed on the Fe_{55} catalyst surface. **b** adhesion energies for different concentrations, here hydrogen is adsorbed at random positions on the catalyst surface. The dashed lines are linear regressions to the data points. **c** variation in adhesion energies with respect to the position of the adsorbed hydrogen, randomly distributed over the surface (random), close to the tube-catalyst interface (interface) and far away from the interface (bottom).

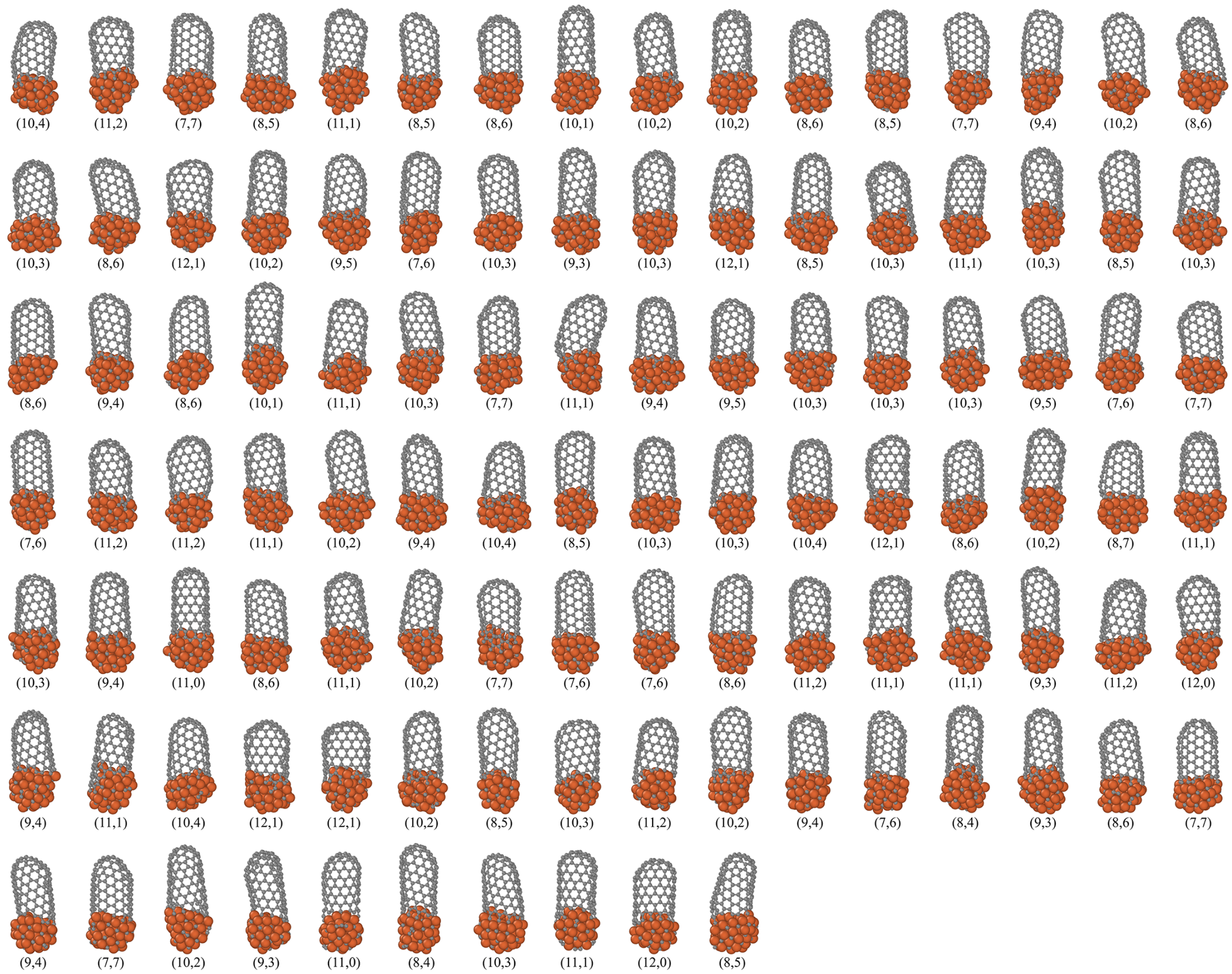

Fig. S7. The structure of the 106 single-walled carbon nanotubes (SWCNTs) with well-defined chirality obtained after growth and analyzed in Fig. S3. The chirality (n, m) of each tube is marked below.

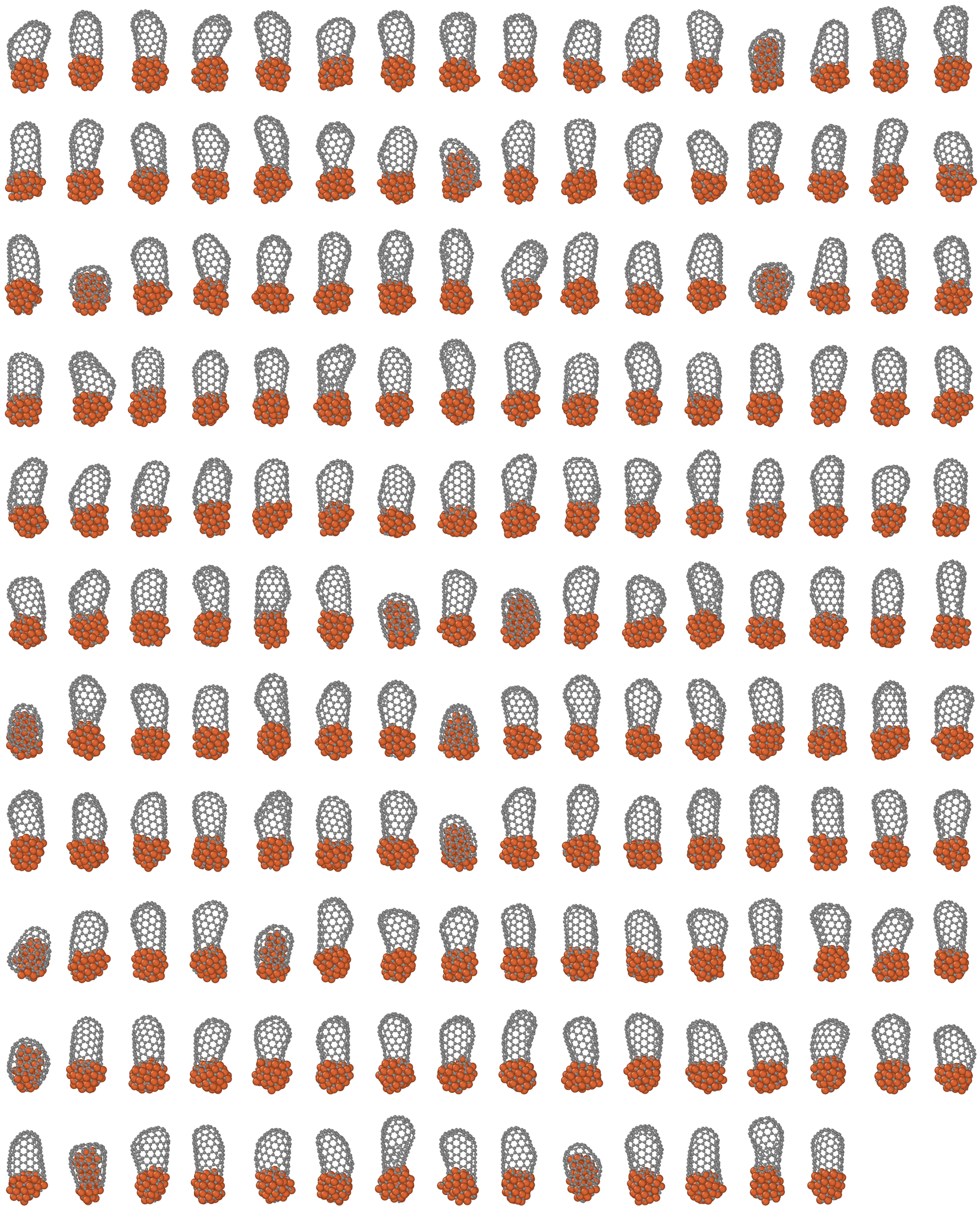

Fig. S8. The structure of the 174 single-walled carbon nanotubes (SWCNTs) with undefinable chirality obtained after growth. Here various failure modes can be seen.

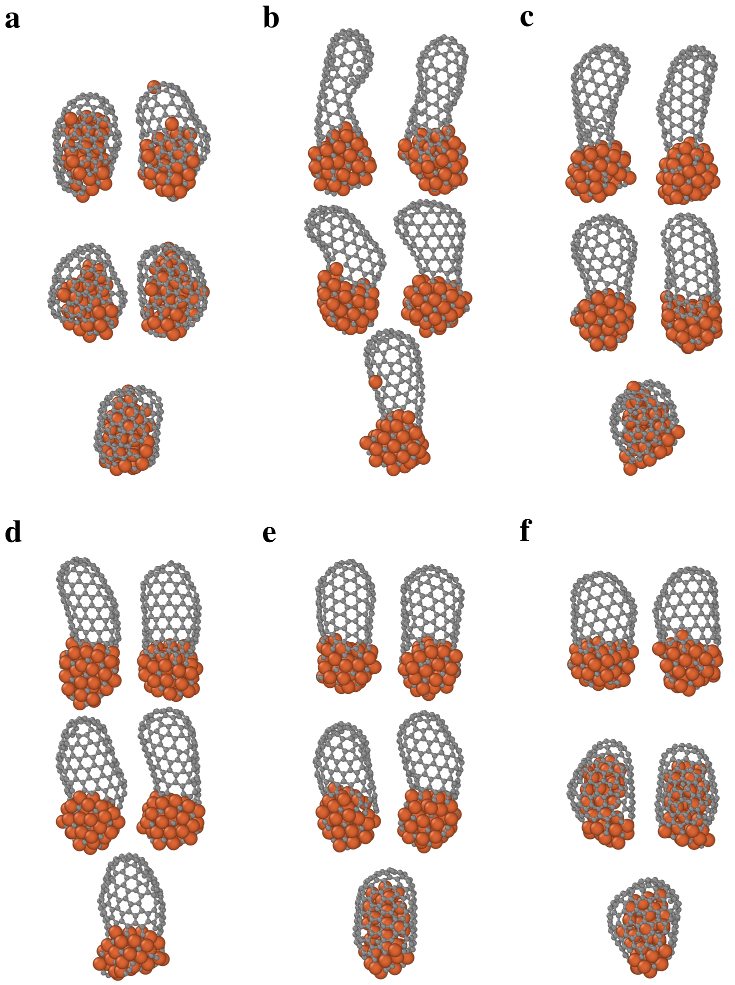

Fig. S9. Effect of temperature on the growth of single-walled carbon nanotubes (SWCNTs). Here tubes are grown on a Fe_{55} catalyst at a carbon supply rate of $k = 1.0$ ns^{-1}. 5 simulations were performed for each growth temperature, T, **a** 1000 K, **b** 1100 K, **c** 1200 K, **d** 1300 K, **e** 1400 K and **f** 1500K.

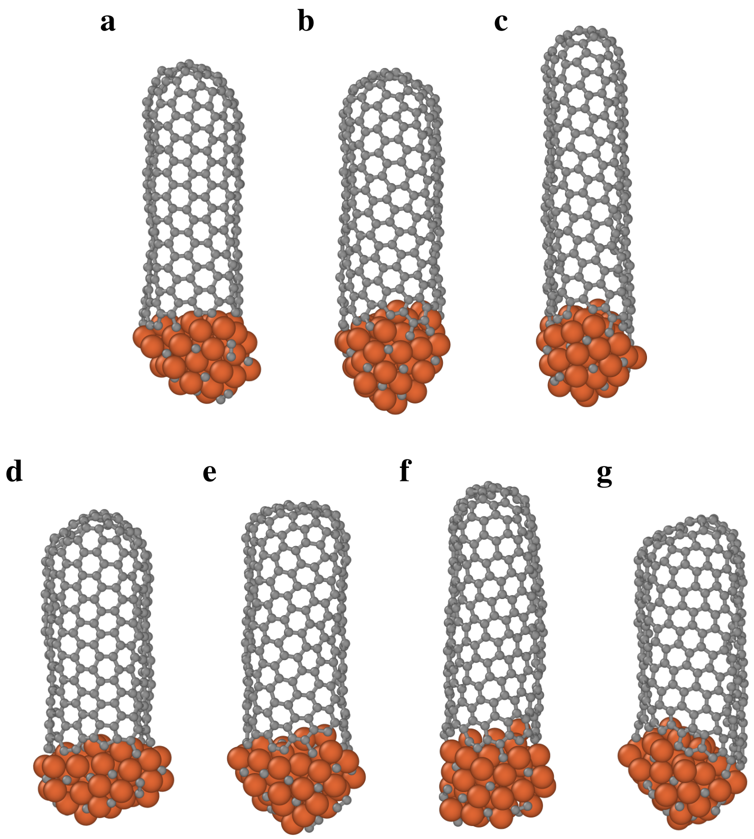

Fig. S10. Additional long defect-free single-walled carbon nanotubes (SWCNTs) grown on a Fe_{55} catalyst. Here **a** is a $(7,7)$ SWCNT grown over 598 ns at $T = 1300$ K and a rate of $k = 0.5$ ns^{-1}. **b** $(9,5)$ SWCNT grown over 598 ns at $T = 1300$ K and a rate of $k = 0.5$ ns^{-1}. **c** $(8,4)$ SWCNT grown over 303 ns at $T = 1300$ K and a rate of $k = 1.0$ ns^{-1}. **d** $(8,7)$ SWCNT grown over 598 ns at $T = 1500$ K and a rate of $k = 0.5$ ns^{-1}. **e** $(9,5)$ SWCNT grown over 303 ns at $T = 1500$ K and a rate of $k = 1.0$ ns^{-1}. **f** $(11,2)$ SWCNT grown over 303 ns at $T = 1500$ K and a rate of $k = 1.0$ ns^{-1}. **g** $(12,2)$ SWCNT grown over 303 ns at $T = 1500$ K and a rate of $k = 1.0$ ns^{-1}.

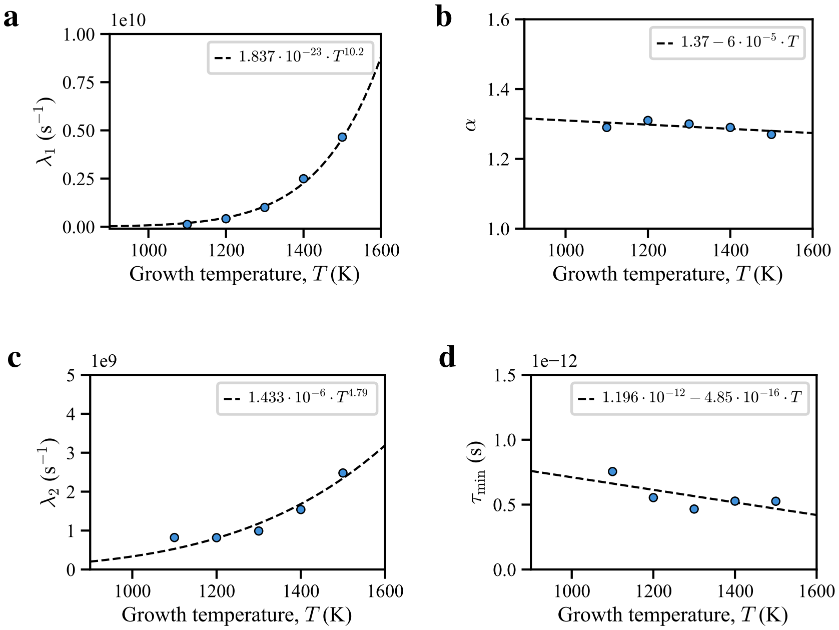

Fig. S11. The temperature dependence for the parameters in Eq. (1) (**a**: λ_1) and Eq. (2) (**b**: α, **c**: λ_2 and **d**: τ_{min}) of the main text. Here the data points are obtained from fitting Eq. (1) and Eq. (2) of the main text to the normalized cumulative sum of the measured values of δ_t and τ from Sim. 2, 3, 4, 5 and 6 outlined in the main text, Table 1.

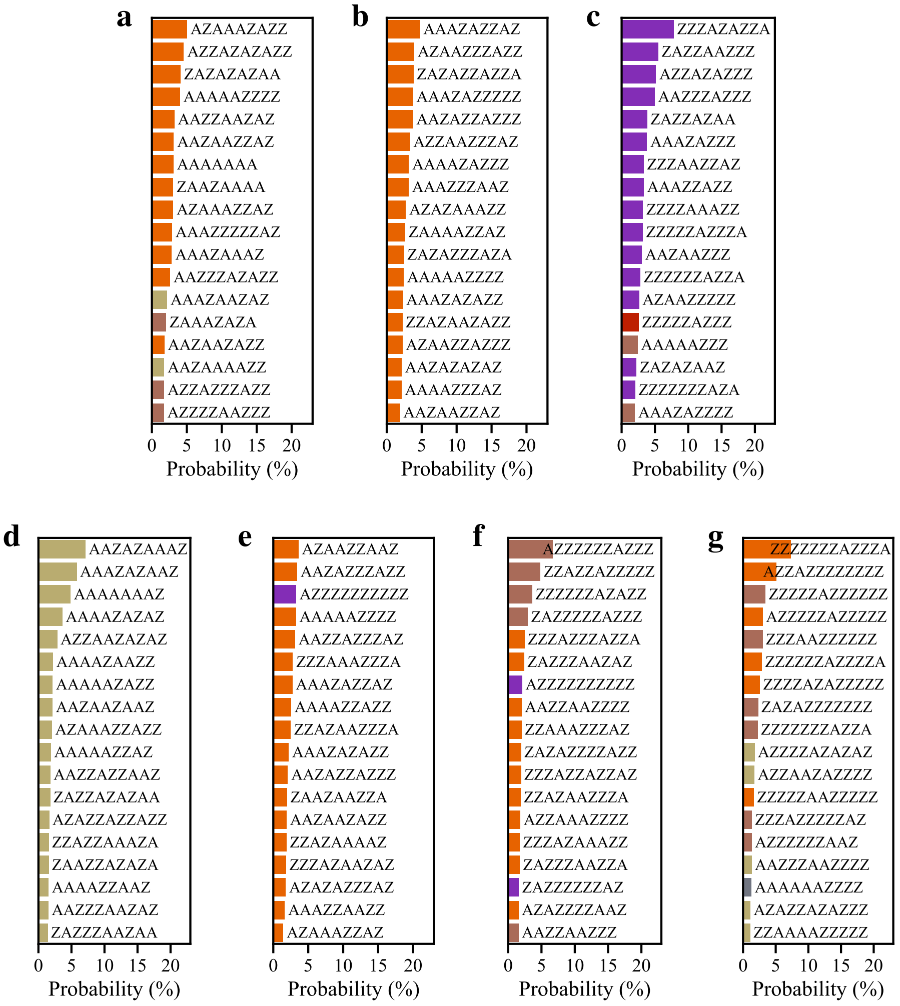

Fig. S12. The 18 most observed edge configurations during growth of the; **a** (7,7), **b** (9,5), **c** (8,4), **d** (8,7), **e** (9,5), **f** (11,2) and **g** (12,2) single-walled carbon nanotubes (SWCNTs) shown in Fig. S10. Here the color of the bars represents the length of the edge where red: 10, grey: 11, purple: 12, brown: 13, orange: 14, tan: 15 and dark grey: 16 atoms. For each bar, the corresponding edge configuration is labeled with zigzag sites denoted by Z and armchair pairs denoted by A. The sample size used to calculate the probabilities were **a** 149,360, **b** 171,279, **c** 72,222, **d** 155,575, **e** 74,247, **f** 58,180 and **g** 54,885 edge configurations.

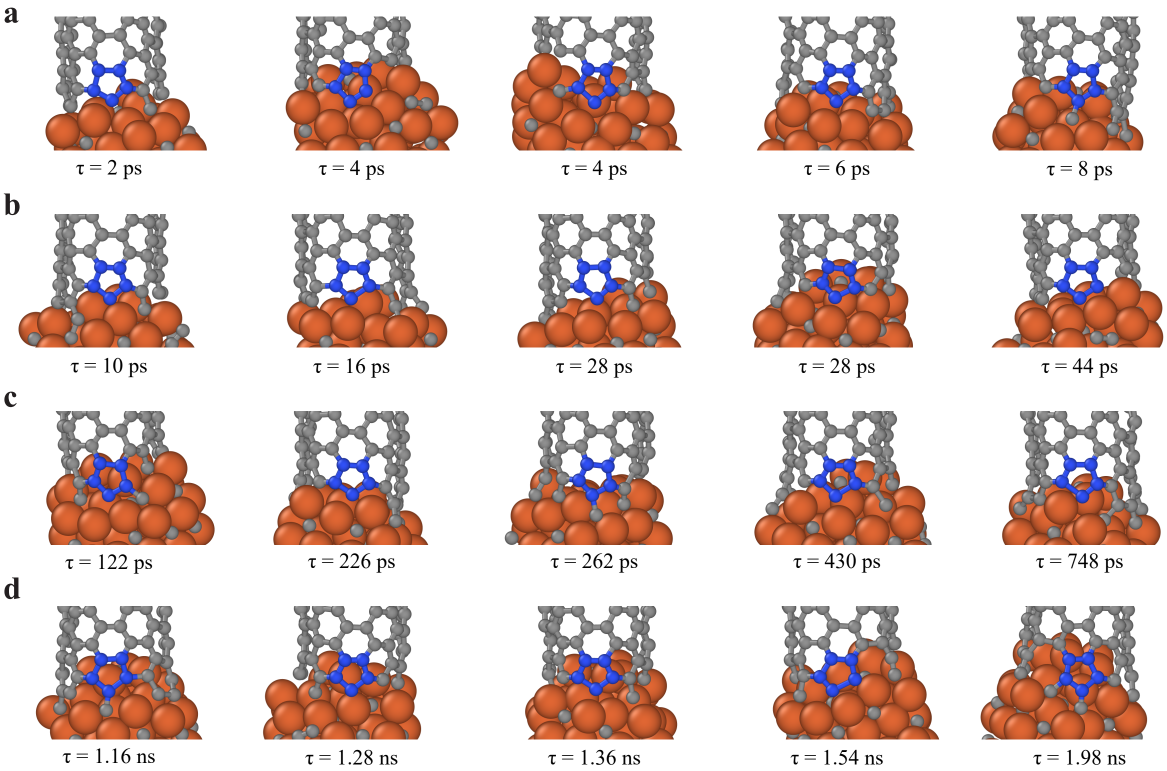

Fig. S13. Interface defect configurations observed during single-walled carbon nanotube (SWCNT) growth on a Fe_{55} catalyst. Here, the structure of several randomly selected interface defects (highlighted in blue), at the time of their formation, are grouped by the order of magnitude of their lifetimes, **a** 10^{-12} s, **b** 10^{-11} s, **c** 10^{-10} s and **d** 10^{-9} s.

References

1. Morrow, J. D., Gardner, J. L. A. & Deringer, V. L. How to validate machine-learned interatomic potentials. *The Journal of chemical physics* **158,** 121501. https://doi.org/10.1063/5.0139611 (Mar. 2023).

2. Zeng, J., Cao, L., Xu, M., Zhu, T. & Zhang, J. Z. H. Complex reaction processes in combustion unraveled by neural network-based molecular dynamics simulation. *Nature communications* **11,** 5713. https://doi.org/10.1038/s41467-020-19497-z (Nov. 2020).

3. Zeng, J., Zhang, L., Wang, H. & Zhu, T. Exploring the Chemical Space of Linear Alkane Pyrolysis via Deep Potential GENerator. *Energy & Fuels* **35,** 762–769. https://doi.org/10.1021/acs.energyfuels.0c03211 (Dec. 2020).

4. Zhang, Y. *et al.* DP-GEN: A concurrent learning platform for the generation of reliable deep learning based potential energy models. *Computer physics communications* **253,** 107206. https://doi.org/10.1016/j.cpc.2020.107206 (Feb. 2020).

5. Kudin, K. N., Scuseria, G. E. & Yakobson, B. I. C_2F, BN, and C nanoshell elasticity from ab initio computations. *Physical Review B* **64,** 235406. https://doi.org/10.1103/PhysRevB.64.235406 (Nov. 2001).

6. Hedman, D. & Larsson, J. A. Analytical modelling of single-walled carbon nanotube energies: the impact of curvature, length and temperature. *SN Applied Sciences* **2.** https://doi.org/10.1007/s42452-020-2139-z (Feb. 2020).

7. Larsson, P. *et al.* Calculating carbon nanotube-catalyst adhesion strengths. *Physical Review B* **75,** 115419. https://doi.org/10.1103/PhysRevB.75.115419 (Mar. 2007).

8. Ding, F. *et al.* The Importance of Strong Carbon-Metal Adhesion for Catalytic Nucleation of Single-Walled Carbon Nanotubes. *Nano Letters* **8,** 463–468. https://doi.org/10.1021/nl072431m (Dec. 2007).

9. Silvearv, F., Larsson, P., Jones, S. L. T., Ahuja, R. & Larsson, J. A. Establishing the most favorable metal-carbon bond strength for carbon nanotube catalysts. *Journal of Materials Chemistry C* **3,** 3422–3427. https://doi.org/10.1039/C5TC00143A (2015).

10. Ding, L. P. *et al.* Why Carbon Nanotubes Grow. *Journal of the American Chemical Society* **144,** 5606–5613. https://doi.org/10.1021/jacs.2c00879 (Mar. 2022).

11. Chiang, W.-H. & Sankaran, R. M. Linking catalyst composition to chirality distributions of as-grown single-walled carbon nanotubes by tuning Ni(x)Fe(1-x) nanoparticles. *Nature Materials* **8,** 882–886. https://doi.org/10.1038/nmat2531 (Sept. 2009).

12. He, M., Jiang, H., Kauppinen, E. I. & Lehtonen, J. Diameter and chiral angle distribution dependencies on the carbon precursors in surface-grown single-walled carbon nanotubes. *Nanoscale* **4,** 7394–7398. DOI%09https://doi.org/10.1039/C2NR32276E (Oct. 2012).

13. Ago, H. *et al.* CVD Growth of Single-Walled Carbon Nanotubes with Narrow Diameter Distribution over Fe/MgO Catalyst and Their Fluorescence Spectroscopy. *The Journal of Physical Chemistry B* **109,** 10035–10041. https://doi.org/10.1021/jp050307q (May 2005).

14. Diaz, M. C., Jiang, H., Kauppinen, E., Sharma, R. & Balbuena, P. B. Can Single-Walled Carbon Nanotube Diameter Be Defined by Catalyst Particle Diameter? *The Journal of Physical Chemistry C* **123,** 30305–30317. https://doi.org/10.1021/acs.jpcc.9b07724 (Dec. 2019).

15. Yang, F. *et al.* Growth modes of single-walled carbon nanotubes on catalysts. *Science advances* **8,** eabq0794. https://doi.org/10.1126/sciadv.abq0794 (Oct. 2022).

16. Ding, F., Rosén, A. & Bolton, K. Molecular dynamics study of the catalyst particle size dependence on carbon nanotube growth. *The Journal of chemical physics* **121,** 2775–2779. https://doi.org/10.1063/1.1770424 (July 2004).

17. Xu, Z. & Ding, F. Catalyst particle size dependent carbon nanotube cloning. *Carbon* **175,** 69–76. https://doi.org/10.1016/j.carbon.2020.12.085 (Apr. 2021).

18. Xu, Z., Qiu, L. & Ding, F. The kinetics of chirality assignment in catalytic single-walled carbon nanotube growth and the routes towards selective growth. *Chemical Science* **9,** 3056–3061. https://doi.org/10.1039/C7SC04714B (2018).
