# Supplementary material for: Dynamics of growing carbon nanotube interfaces probed by machine learning-enabled molecular simulations
Source: Nat Commun. 2024 May 14;15:4076. doi: 10.1038/s41467-024-47999-7 (PMC11094095; doi:10.1038/s41467-024-47999-7)
Supplement: Supplementary file 3 — Description of Additional Supplementary Files [file 41467_2024_47999_MOESM3_ESM.docx]

**Description of Additional Supplementary Files**

**File Name:** Supplementary Movie 1.mp4
**Description:** Visualization of the growth simulation of the (6,5) single-walled carbon nanotube (SWCNT). Atomic-level details are provided for the complete growth process of the (6,5) SWCNT. Here orange and grey spheres represent Fe and C atoms, respectively. Each second of the movie corresponds to 3.84 ns of growth.

**File Name:** Supplementary Movie 2.mp4
**Description:** Visualization of the healing of a pentagon interface defect. Atomic-level details are provided for the healing of a pentagon defect at the tube-catalyst interface during the growth of the (6,5) single-walled carbon nanotube. Here orange and grey spheres represent Fe and C atoms, respectively, and blue spheres depict C atoms initially belonging to a pentagon interface defect. Each second of the movie corresponds to 60 ps of simulation time.

**File Name:** Supplementary Movie 3.mp4
**Description:** Visualization of the healing of a penta-heptagon interface defect. Atomic-level details are provided for the healing of a penta-heptagon pair at the tube-catalyst interface during the growth of the (6,5) single-walled carbon nanotube. Here orange and grey spheres represent Fe and C atoms, respectively, and blue and green spheres depict C atoms initially belonging to a pentagon and heptagon interface defect, respectively. Each second of the movie corresponds to 60 ps of simulation time.

**File Name:** Supplementary Movie 4.mp4
**Description:** Visualization of the nanotube edge during growth. Atomic-level details are provided for the dynamics of the nanotube edge during growth of the (6,5) single-walled carbon nanotube. Here the edge configuration is shown where blue corresponds to zigzag sites, orange armchair pairs and orange-green-orange are directly nucleated hexagons. Each second of video corresponds to 3.84 ns of growth.

**File Name:** Supplementary Data 1.xyz
**Description:** The density functional theory relaxed structures generated in this study. The first 8 structures are the (6,6) single-walled carbon nanotube (SWCNT) attached to the Fe_55_ cluster with different amounts of hydrogen adsorbed. The last 8 structures are the (10,0) SWCNT attached to the Fe_55_ cluster with different amounts of hydrogen adsorbed. The structures are given in the xyz format.
